# Commentary: Do Bees Play the Producer-Scrounger Game?

**DOI:** 10.3389/fpsyg.2016.01355

**Published:** 2016-09-07

**Authors:** Mathieu Lihoreau, Cristian Pasquaretta, Philipp Heeb

**Affiliations:** ^1^Research Center on Animal Cognition, Center for Integrative Biology, Centre National de la Recherche Scientifique, UPS, University of ToulouseToulouse, France; ^2^Laboratoire Évolution et Diversité Biologique, UMR 5174 Centre National de la Recherche Scientifique, Université Paul SabatierToulouse, France

**Keywords:** bumblebees, social learning, producer-scrounger game, social foraging, bumble bees, social cognition

Group-living animals often use social information, in addition to personal sampling, to learn about foraging opportunities. Small-brained insects are no exception (Grüter and Leadbeater, 2014). For instance, inexperienced bumblebees learn to identify profitable flower species by observing conspecifics (Leadbeater and Chittka, 2005). Bumblebees are especially suitable to study insect social learning as they can be easily tested in the lab, allowing for precise control of food resources, individual experience and social cues (Avarguès-Weber et al., 2015). Here we comment on two recent studies showing how bumblebees use personal and social information discriminately to make adaptive foraging decisions, thus setting the scene for complex social foraging dynamics among bees exploiting variable ressources in the field.

Writing in *Current Biology*, Dunlap et al. (2016) report that foragers of the common eastern bumblebee *(Bombus impatiens*) rely more on social information than on personal information if the former predicts a reward. The authors trained bumblebees to collect sucrose solution in arrays of 12 artificial flowers in which they manipulated personal information (by using yellow or orange flowers) and social information (by marking some flowers with a pinned dried conspecific). During training, each type of information was either fully reliable (100% of flowers rewarded), moderately reliable (83% of flowers rewarded), or unreliable (50% of flowers rewarded) in a full factorial design of nine treatments. Bumblebees were equally successful at associating personal and social information to a reward. However during the test, in which only unrewarded flowers of all four types were used, most bumblebees preferred flowers with a conspecific. For treatments in which social information was moderately or highly reliable, bumblebees always preferred flowers with a conspecific, even if personal information was more reliable. When both information were unreliable, bumblebees did not show any preference. Only, when social information was unreliable and personal information moderately or highly reliable, bumblebees preferred flowers without a conspecific.

In another study published in *Biology Letters*, Smolla et al. (2016) show that buff-tailed bumblebees (*Bombus terrestris*) rely on social information when personal information is unreliable. The authors developed an evolutionary agent-based model to predict conditions when personal information should be favored over social information with resources equally distributed among flowers (low uncertainty), whereas social information should be favored when resources vary across flowers (high uncertainty). This prediction was tested by training bumblebees to collect rewards from 12 artificial flowers while successively manipulating personal information (by varying the distribution of rewards in flowers) and social information (by marking flowers with clay model bees). Bumblebees were first trained to associate social information to a reward on colorless flowers. Four flowers were marked with a model bee and two of them contained a reward. The same bumblebees were then trained to assess environmental certainty by foraging on yellow flowers that were either all rewarded (low uncertainty) or only two were rewarded (high uncertainty). When tested with 12 unrewarded flowers (8 yellow without conspecifics, 4 yellow with conspecifics), bumblebees trained in the high uncertainty condition preferred flowers with conspecifics whereas those trained in the low uncertainty condition did not. Interestingly, this “copy-when-uncertain strategy” was not observed when model bees were replaced by green rubber foam, suggesting selection for sensory and/or learning biases toward social cues.

While facultative social information use by insects has been previously described (Grüter and Leadbeater, 2014), these two studies demonstrate a novel level of sophistication, suggesting the occurrence of more complex social foraging dynamics in populations of freely interacting bees, where the value of personal and social information may vary dynamically with resource availability, social, and competitive interactions (Lihoreau et al., 2016). In vertebrates, social foraging decisions have long been framed in terms of the producer-scrounger (PS) game, in which foragers have the options to either search for resources (producers) or exploit the efforts of others (scroungers) to obtain resources (Barnard and Sibly, 1981; Galef and Giraldeau, 2001). Three conditions must be met for the PS game to take place (Morrand-Ferron et al., 2007): (1) foraging strategies must be exclusive (an individual cannot simultaneously be producer and scrounger), (2) this exclusivity generates a negative frequency dependence of the payoffs to scroungers, (3) group-level adjustments to variation in socio-ecological factors determine the stable equilibrium frequency of scroungers.

Despite growing interest in insect social learning abilities, tests of the PS game in invertebrates are still surprisingly scant (Dumke et al., 2016). Here we argue that bumblebees have the behavioral and cognitive requirements to play the PS game. First, bumblebee foragers show two distinct behaviors by either personally sampling flowers (producers) or joining conspecifics (scroungers), and each individual can be engaged in only one at a given time (Leadbeater and Chittka, 2005). Second, even when the reward value of flowers is fully predictable and bees should use personal information, a non-negligible proportion of foragers continue to follow social information (20% in Smolla et al., 2016), suggesting that scrounging provides frequency dependent benefits that remain to be quantified. Finally, varying the costs of sampling flowers and joining conspecifics produces populations with different proportions of individuals relying on personal and social information (Dunlap et al., 2016). Although this is not a direct demonstration, it suggests that context-dependent shifts in frequencies take place between the two strategies, as expected in a PS game.

Future explorations of the applicability of the PS game in bees should quantify the changes in the frequency-dependent payoffs of producer and scrounger strategies and their variation under changing environmental conditions. Detailed studies of the foraging decisions of freely interacting bees are now becoming possible using automated approaches for tracking individual movements and mapping the influence of information flow on flower choices [e.g., motion detection cameras (Lihoreau et al., 2012), radio frequency identification (Ohashi et al., 2008), computer vision (Crall et al., 2015), network statistics (Pasquaretta et al., 2016)]. Other insects use personal and social information for choosing foods (Foucaud et al., 2013), suggesting that the applicability of the PS game in this group is more general. The hope is to promote the existing theory to further bridge research on vertebrate and invertebrate social cognition.

## Author contributions

All authors listed, have made substantial, direct and intellectual contribution to the work, and approved it for publication.

## Funding

This work is part of the “Laboratoire d'Excellence (LABEX)” entitled TULIP (ANR-10-LABX-41). Additionally, ML receives funding from the Fyssen Foundation, the IDEX of the Federal University of Toulouse and the ANR. CP is funded by the IDEX Emergence program POLLINET to ML.

### Conflict of interest statement

The authors declare that the research was conducted in the absence of any commercial or financial relationships that could be construed as a potential conflict of interest.
